# Dietary and Nutrient Intake, Eating Habits, and Its Association with Maternal Gestational Weight Gain and Offspring’s Birth Weight in Pregnant Adolescents

**DOI:** 10.3390/nu14214545

**Published:** 2022-10-28

**Authors:** Reyna Sámano, Hugo Martínez-Rojano, Luis Ortiz-Hernández, Oralia Nájera-Medina, Gabriela Chico-Barba, Estela Godínez-Martínez, Ricardo Gamboa, Estefanía Aguirre-Minutti

**Affiliations:** 1 Programa de Posgrado Doctorado en Ciencias Biológicas y de la Salud, División de Ciencias Biológicas y de la Salud, Universidad Autónoma Metropolitana, Mexico City 04960, Mexico; 2 Coordinación de Nutrición y Bioprogramación, Instituto Nacional de Perinatología, Secretaría de Salud, Mexico City 11000, Mexico; 3 Sección de Posgrado e Investigación de la Escuela Superior de Medicina del Instituto Politécnico Nacional, Mexico City 11340, Mexico; 4 Departamento de Atención a la Salud, CBS, Universidad Autónoma Metropolitana-Xochimilco, Mexico City 04960, Mexico; 5 Programa de Maestría y Doctorado en Ciencias Médicas, Odontológicas y de la Salud, Universidad Nacional Autónoma de México, Mexico City 04510, Mexico; 6 Departamento de Fisiología, Instituto Nacional de Cardiología, Mexico City 14080, Mexico; 7 Licenciatura en Nutrición, Centro Interdisciplinario de Ciencias de la Salud, Unidad Milpa Alta (CICS), Instituto Politécnico Nacional, Mexico City 12000, Mexico

**Keywords:** adolescent pregnancy, gestational weight gain, energy intake, food groups, dietary habits, Mexico

## Abstract

Pregnant adolescents’ diet and eating habits are inadequate; however, their association with gestational weight gain (GWG) is uncertain. We aimed to analyze whether there is an association between dietary and nutrient intake and eating habits with GWG among pregnant adolescents and their offspring’s birth weight. A longitudinal study was performed with 530 participants. We assessed GWG and applied several tools, such as a food frequency questionnaire and 24-h recall, to obtain dietary and nutrient intake and eating habits. The birth weight of adolescents’ offspring was registered. Later, we performed crude and adjusted Poisson models. The mean age was 15.8 ± 1.3 years. Of all food groups, the lowest frequency of adequate intake corresponded to vegetables (7%) and legumes (10.2%). Excessive (36.8%) and insufficient (40.9%) GWG were observed. Pregnant adolescents with inadequate legumes intake increased the probability of excessive GWG: (PR 1.86 95% CI 1.00–3.44). Cereals and grains were positively associated with GWG: (PR 1.65, 95% CI 1.18–2.29). Energy, macronutrient intake, and eating habits were not associated with GWG. Offspring’s small gestational age (SGA) increased when pregnant adolescents had inadequate sugar-sweetened beverages intake: PR (1.58, 95% CI 1.01–2.49) and when pregnant adolescent watched television (TV). In our sample of Mexican adolescents, dietary and nutrient intake and eating habits were inadequate. Excessive dietary intake from cereals, grains, and animal-sourced foods along with insufficient legumes were associated with excessive GWG. Watching TV while adolescents ate was associated with the birth weight of the offspring.

## 1. Introduction

Adolescent pregnancy represents a global public health concern. Nearly 20% of adolescents from low and middle-income countries give birth [1,2]. They have a higher frequency of adverse outcomes such as preterm birth, small-for-gestational-age (SGA), and increased neonatal and maternal mortality risk than pregnant adults [3,4,5]. Gestational weight gain (GWG) has been associated with both short-term and long-term consequences, such as anemia and preeclampsia. In the short-term, excessive GWG is associated with adverse newborn outcomes, including preterm birth, large-for-gestational-age, and macrosomia. In the long term, it is associated with significant weight retention after pregnancy and excess body weight later in the mother’s life [6]. Therefore, pregnant adolescents need more health services, which are associated with higher costs to provide them with prenatal and postnatal care [7,8]. In addition, although pregnant adolescents have a similar proportion of excessive gestational weight gain (GWG) compared to adults, the former have a higher total GWG in kilograms (kg) [6].

Several countries in sub-Saharan Africa, Latin America, and Asia have moved from low-income to middle-income status, which is accompanied by lifestyle changes, including increased food security, dietary transitions, and reduced physical activity. These changes have led to modifications in maternal diets before and during pregnancy, affecting GWG patterns and the overall pregnancy experience for women in these regions [9,10,11]. For example, a study from Tanzania reported that, according to Institute of Medicine (IOM) guidelines, 42.0%, 22.0%, and 36.0% of pregnant adults were characterized as having inadequate, adequate, and excessive GWG, respectively [12].

Another problem is that, as pregnant adolescents’ linear growth has not reached its peak, their nutrient requirements are higher than adult women [13]. Nevertheless, studies about the dietary patterns of pregnant adolescents are scarce [14]. Pregnant adolescents tend to have low iron intake (28% for Recommended Dietary Allowances-RDA) [15,16]. Moreover, less than 30% have good adherence to folate supplementation [15]. The average intake of calcium in pregnant adolescents from the USA [16], Brazil, and Mexico [15,17,18] ranges from 400 to 900 mg/day, which does not meet the recommended intake of 1000–1300 mg/day [19]. This inadequate nutrient intake in pregnant adolescents can be linked to the low variety of food groups they consume [20,21]. At least 75% of pregnant adolescents who received antenatal care in a public hospital had low intake of vegetables and legumes, around 50% consumed more sweetened beverages than recommended, and 5–25% skipped supper or breakfast [22].

Dietary and nutrient intake and eating habits can potentially affect GWG, as energy and nutrients are necessary for tissue accretion [23]. A few studies have been conducted on adult women to analyze that relationship [24,25,26]. However, systematic reviews about this topic included only adult women [27,28,29]. We could not find any studies on the association of dietary and nutrient intake and eating habits with GWG and offspring’s birth weight in pregnant adolescents [29]. Nevertheless, evidence derived from Rumanian adult women showed a positive association between a high-fat diet and excessive GWG and a negative association with a high-protein diet [26]. In addition, adult pregnant women who consume foods from the Mediterranean diet (legumes, vegetables, nuts, olive oil, and whole cereals) have high odds of having a lower [24] or adequate GWG [24,25] and a lower risk of having a small-for-gestational-age newborn when eating fruits and vegetables [30,31,32]. Energy intake has been associated with GWG, while macronutrients have not [28]. This paper aimed to analyze whether there is an association between dietary and nutrient intake and eating habits and GWG among pregnant adolescents and their offspring’s birth weight.

## 2. Materials and Methods

We conducted a longitudinal study with pregnant adolescents aged 11–19 years who received antenatal care at the Instituto Nacional de Perinatología (INPer) in Mexico City. The inclusion criteria were being a woman primigravida with single pregnancy and without chronic diseases. In addition, adolescents with drug addictions, vegans or vegetarians, and those who had a newborn with congenital malformations or stillbirth were excluded.

Six hundred and fifty adolescents were invited to participate in the study. Forty teenagers did not agree to participate, 38 accepted but did not arrive at any assessment, 25 did not deliver at the INPer, 15 cases were incomplete, and two neonates died at birth. There were 530 cases with complete data. During the first visit, we obtained signed consent from adolescents and their parents/guardians as well as sociodemographic data. Anthropometric measurements and dietary assessment were conducted. We obtained maternal and neonatal outcomes from the last consultation from the medical records.

### 2.1. Dietary and Nutrient Intake, and Eating Habits

We assessed food group consumption to describe dietary intake using a semi-quantitative food frequency questionnaire (FFQ) [33]. Intake of nine food groups was measured. The dietary guidelines for the Mexican population were used as criteria. These guidelines present the following food groups: vegetables; fruits; legumes; cereal and grains; meat, cheese, and eggs (herein, “animal-source foods”); fats and oils; milk and yogurt; table sugar; and sweetened beverages [34]. Participants reported their frequency of intake during the last trimesters. Because it is known that macronutrient intake remains relatively stable during pregnancy [35], one measurement in the second or third trimesters was obtained. The interviewers used food replicas and standard measuring cups, spoons, and glasses to improve serving size estimation. Later, we compared the number of servings consumed with the recommendations for the Mexican population [34]. The number of servings of each food group used as a reference can be reviewed in Appendix A. Adequate consumption was defined when the number of servings was met according to the recommendation. Inadequate consumption (excessive and insufficient) was when the participants ate more or fewer servings than the recommendation range.

Three 24-h dietary recalls were applied. Two were recorded on non-consecutive weekdays and another on weekends. The 24-h recalls were administered by personnel trained in the interview technique. The nutrient and energy intake were estimated using Nutrikcal^®^ software. Later, the mean energy intake in kilocalories (kcal) was calculated. To measure participants’ energy intake adequacy, we used the reference of the IOM (2005) [36,37]. We categorized energy intake adequacy as insufficient (<80%), adequate (80–119%), or excessive (>120%). The contribution of carbohydrates, proteins, and lipids to total energy consumption was estimated. The recommendations of the IOM were used as a reference to categorize the distribution of energy contribution of macronutrients [37].

Participants were asked about the following eating habits: their number of meals; frequency of skipping meals (never, 1–3 times, 4–5 times/week); with whom they ate their foods (alone, with family, and friends); where they ate (out of home, home); and what activities they did while eating (doing homework/household chores, watching TV or using a cellphone, or just eating). In addition, we inquired as to whether participants had modified their diet during pregnancy (if it was improving, was worse, or had no change).

### 2.2. Anthropometric Data and Gestational Weight Gain

In the first interview, the pre-pregnancy self-reported weight was obtained. The self-reported weight is an adequate proxy for pre-pregnancy weight [38,39].

All anthropometric measurements were performed according to Lohman’s techniques [40]. Height was measured at the first antenatal visit using a stadiometer (SECA, Hamburg, Germany, model 208, accuracy 0.1 cm). We estimated the pregestational body mass index (pBMI) using the pregestational weight and height. Then, we classified pBMI with AnthroPlus^®^ (World Health Organization, Geneva, Switzerland) according to percentiles: underweight <3rd, normal weight 3–85th, overweight 85–97th, and obesity ≥97th [41].

One or two weeks before delivery, we measured and recorded participants’ body weight with a digital scale (TANITA, Tokyo, Japan, model BWB-800, accuracy 0.10 kg). This measure was considered the final gestational weight. The GWG was calculated from the difference between the final gestational weight and the pregestational weight.

The expected weight gain was calculated with the following equation [42]:

Expected weight gain = recommended weight gain for the first trimester + ((gestational age final—13.86 weeks) × (recommended weight gain rate in second and third trimesters)).

The recommendation of GWG rate for the first trimester was according to pBMI: low and normal weight 2 kg, overweight 1 kg, and obesity 0.5 kg. For adolescents in the second and third trimesters, these pBMI figures were low weight 0.51 kg, normal weight 0.42 kg, overweight 0.28 kg, and obesity 0.22 kg/week [43].

The gestational weight gain adequacy percentage was estimated using the recommendations of the US Institute of Medicine [43,44]. Finally, we categorized the GWG percentage as follows: inadequate (<90%), adequate (90 to <125%), and excessive (≥125%).

### 2.3. Neonatal Outcomes

The sex of the newborn was obtained from the neonatal clinical record. Gestational age was obtained by ultrasound and recorded in weeks and days. If the gestational age was ≤36.6 weeks we classified it as preterm, whereas if the gestational age was between ≥37 and ≤42 weeks this was considered at term.

Standardized personnel measured and recorded birth weight (g) with calibrated equipment (SECA 374, model “Baby and Mommy”; accuracy 0.1 g) and length at birth (cm) (stadiometer SECA 416; accuracy 0.1 cm). SGA was defined when birth weight was <10 percentile, normal birth weight as the neonate being between 10–90 percentile, and large for gestational age (LGA) as >90 percentile, according to the Intergrowth-21s criteria [45].

### 2.4. Other Variables

In an antenatal visit, trained personnel obtained information on sociodemographic characteristics such as chronological age, marital status, education, occupation, and socioeconomic level. Age was registered at the time of the survey in years and as a dichotomous variable (≤15 or ≥16 to 19 years). In addition, marital status was classified as cohabiting or single.

Education was reported by the pregnant adolescents and was considered as elementary school or less, middle school, and incomplete high school. In addition, we created a school dropout variable according to the school grade and chronological age for adolescents who were more than two years behind in educational training.

Occupation was classified as student or housewife. A questionnaire validated for the Mexican population was used to determine socioeconomic status [46]. In our sample only middle, low–middle, and low were observed.

The initiation of antenatal care and the gestational age at delivery were obtained through ultrasound and reported in weeks. Obstetricians registered maternal adverse outcomes during prenatal visits, and the information from the clinical records was obtained. Complications were identified and recorded in the following categories: gestational diabetes, pregnancy-induced hypertension, eclampsia/pre-eclampsia, and anemia [47,48].

### 2.5. Statistical Analyses

A descriptive analysis was performed, including percentages for categorical variables. For continuous variables, the Kolmogorov–Smirnov test was used to assess their distribution. The mean was estimated for variables with normal distribution, and the median was obtained for those with a non-normal distribution. Next, we compared the prevalence of outcomes between the categories of nutrition, energy intake, and eating habits. The chi-square test was estimated to assess whether significant differences between categories existed. When the significance of the difference was *p* ≤ 0.250, the variable was considered for the next step.

Poisson regression models were calculated to determine the association of outcomes (GWG and offspring’s birth weight) with predictors of interest (nutrients, energy intake, and eating habits). We estimated separate models for inadequate and excessive GWG. For this reason, dummy variables were created for the GWG and birth-weight categories. For each outcome, three models were performed: M1, crude M2, adjusted by socioeconomic level, school drop-out, education, gynecological age, chronological age, and antenatal care; and M3, adjusted by the same variables included in M2 plus pBMI. The regression coefficients were transformed to prevalence ratios (PR).

When the cross-tabulation of two eating habits with the outcomes was estimated, the absence of any cases in certain cells was evident. Hence, these variables were not included in the regression analysis.

### 2.6. Ethical Aspects

This research was approved by the Institutional Ethics, Biosafety, and Research Committees from INPer (registration numbers 212250-49481, 212250-49541, and 2017-2-101, respectively). All adolescents and their guardians were informed of the study’s objectives and procedures. Confidentiality was guaranteed by assigning an ID number during each participant’s data collection and analysis. Written informed consent was obtained from adolescents and guardians.

## 3. Results

The mean age of the participants was 15.8 ± 1.3 years. Seventy percent of the adolescents were single, and the rest lived cohabiting with their partners. Most adolescents were homemakers (89%). Their socioeconomic status was low or very low. Three-quarters of the women had elementary education (74.7%). School dropout was experienced by 89.1%.

Gestational weight gain in pregnant adolescents was excessive in 36.6%, adequate in 26%, and insufficient in 37.4%. In addition, it was observed that 20.4% of newborns were SGA (<10th percentile) and 3.8% were LGA (>90th percentile).

**Figure 1:**
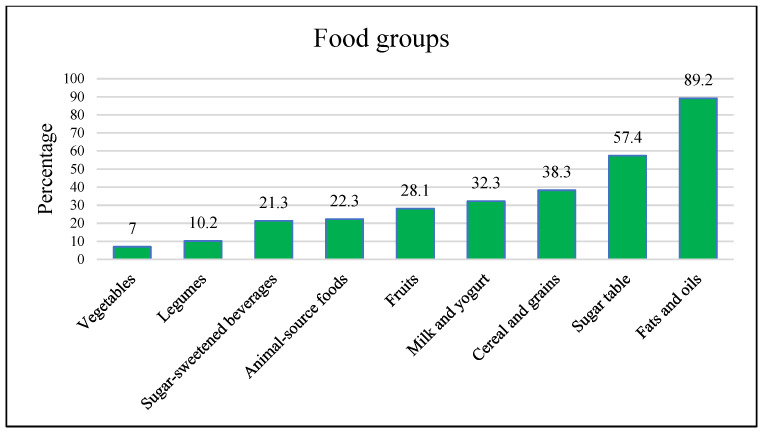
Distribution of adequate intake from different food groups.

The lowest frequency of adequate intake corresponded to vegetables, followed by legumes and animal-source foods (Figure 1). In contrast, the food groups that were eaten most frequently were table sugar, cereals and grains, and dairy foods. None of the nine food groups reached 50% recommended consumption coverage. In addition, 73% of the participants included less than three food groups in their diet.

One-fifth of the adolescents had two or less meals. Dinner was the most skipped meal. Fifty-six percent skipped meals more than once a week. Fifty-one percent of adolescents watched TV while they ate, and 66% reported that their diet was better during pregnancy than pregestational (Appendix B).

Excessive GWG was more frequent among pregnant adolescents who did not consume legumes than those who consumed them (*p* = 0.023) (Table 1). The adolescents with high consumption of cereals and grains and animal-source foods had a higher frequency of excessive GWG (*p* ≤ 0.001) than those with low or normal consumption. Excessive and insufficient GWG were observed more frequently among pregnant adolescents who excessively consumed sugar-sweetened beverages compared to their counterparts who consumed them adequately (*p* = 0.030). The rate of small for gestational age neonates among mothers who consumed excessive sugar-sweetened beverages was higher than in those with a low intake (*p* = 0.066).

The energy intake of the participants was 2022 ± 657 kcal. The distribution of macronutrients of total energy was as follows: 102 ± 34% energy adequacy, 53 ± 8% carbohydrates, 16 ± 5% proteins, and 31 ± 8% lipids. Table 2 shows that none of the macronutrients and energy intake had statistical significance with respect to the maternal GWG and the birth weight of their offspring.

The frequency of GWG and the newborn weight categories did not differ according to eating habits (Table 3).

Pregnant adolescents with insufficient consumption of legumes had a greater probability of excessive GWG than participants with adequate intake (Table 4). Insufficient consumption of cereals and grains was associated with a higher probability of insufficient GWG. In contrast, the excessive consumption of cereals and grains demonstrated a high probability of excessive GWG. In addition, excessive sugar-sweetened beverage consumption was associated with a higher probability of having a small-for-gestational-age newborn.

Lipids intake and eating habits did not have any association with GWG or newborn weight (Table 5).

## 4. Discussion

The results of the present research show that unhealthy eating habits and nutrient intake are frequent in pregnant adolescents. The participants in our study had excessive intake of cereal and grains, animal-source foods, table sugar, and sugar-sweetened beverages, and insufficient consumption of legumes and vegetables. For example, most did not consume the recommended servings of vegetables (93.0%), legumes (89.8%), or sugar-sweetened beverages (79.8%), among other foods, and showed poor eating habits such as skipping meals (56%), eating alone (20.1%), and carrying out activities (61.3%) while they ate.

The present study showed associations between insufficient legumes and excessive cereal and grains consumption and excessive GWG. Meanwhile, sugar-sweetened beverages consumption and using cell phones/watching TV while eating had associations with birth weight.

### 4.1. Dietary and Nutrients Intake and Eating Habits

Although most of our participants (67%) reported that their diet had improved during the pregnancy, they did not have adequate dietary and nutrient intake or eating habits. Our participants’ dietary intake was low in legumes and vegetables and excessive in sweetened-sugar beverage consumption, which is common in most age groups [21,22,49,50,51]. More than 70% of pregnant adolescents did not eat more than three food groups in their meals. Only fifty percent of Mexican pregnant adolescents in the present study had adequate consumption of energy and macronutrients; similar data has been found in pregnant adults [50]. This dietary pattern could be a risk factor for developing non-transmissible chronic diseases [52,53] and micronutrients deficiencies [54].

More than half of the participants skipped meals, watched TV, or used cell phones while eating. Youth exposed to screens habitually consume ultra-processed foods [55]. Watching TV has been associated with the development of excess weight, obesity, and cardiometabolic risk in the adolescent population [55,56].

### 4.2. Gestational Weight Gain

Our study reported that excessive gestational weight gain in adolescent pregnant women occurred in 36.6% and was insufficient in 37.4%. This highlights that there is currently a higher probability in pregnant adolescents of not meeting the recommendations GWG of the IOM. This is similar to the findings of Santos et al. in adolescent Brazilians, which showed 37% and 33% insufficient and excessive GWG, respectively [57]. A significant rate of insufficient and excessive GWG was observed in our sample of pregnant adolescents. This population likely experiences nutritional transitions and reduced physical activity [58], which may lead to changes in maternal diets before and during pregnancy, thereby affecting GWG patterns [59].

### 4.3. Dietary and Nutrient Intake, Eating Habits, and GWG

Insufficient intake of legumes was associated with a higher risk of excessive GWG, even after adjusting for pBMI. Among pregnant adults from Spain and South Africa, the consumption of diets that include legumes [24,60] has been associated with lower GWG. Legumes have nutrient content (high in fiber and antioxidants but low in fat) that can help with keeping a healthy weight [60]. Nevertheless, there is little information on this topic in adolescent pregnant women.

We observed that a higher intake of cereal and grains was associated with excessive GWG. However, with animal-source foods the association was lost when the models were adjusted for pBMI, showing that GWG was affected more by pBMI than by diet in our group of pregnant adolescents. In addition, it has been documented that pBMI is a better predictor of GWG than other variables such as food consumption [61].

Watching TV is a risk factor for developing obesity [56] because it is a sedentary behavior related to higher consumption of ultra-processed foods. Our study found that adolescents who ate while watching TV were associated with LGA neonates. The mechanisms that explain this relationship may be related to maternal consumption of foods with high energy density [62].

We did not find an association between macronutrients and GWG. Our study coincides partially with a previous systematic review that reported macronutrient intake to not be associated with GWG [28]. Hence, it is challenging to estimate macronutrients, which could affect the possible association between GWG and the birth weight of adolescent’s offspring. Nevertheless, the scientific evidence establishes that a whole diet and the foods that make it up can be more relevant than individual nutrients to GWG [24,25]. In this sense, in the present study, we observed that legumes, cereals, and grains were associated with GWG. However, not all foods or macronutrients were associated with GWG.

### 4.4. Dietary Intake, Eating Habits, and Birth Weight

Sugar-sweetened beverages consumption was associated with SGA. There is insufficient evidence to identify possible causal mechanisms to explain the association between maternal consumption of sugar-sweetened beverages and birth weight outcomes [63,64,65]. Therefore, our findings should be interpreted with caution. However, we believe that an inadequate maternal diet is likely to be associated with the birth weight of their offspring [66].

None of the maternal nutrient intakes were associated with birth weight in our sample, similar to GWG. Data from observational studies indicate that certain dietary habits and patterns during pregnancy have no consistent associations with birth weight. Maternal lack of all foods in their diet is relevant, as it has been demonstrated that the whole diet, beyond individual nutrients, can influence birth weight. However, if most participants do not meet a recommended diet the birth weight effect would likely be attenuated, as reported in pregnant adults [67]. Nonetheless, we did not find scientific evidence to support this hypothesis in the studied group of pregnant adolescents

### 4.5. Limitations and Strengths

Using the IOM references, we observed a high frequency of excessive and insufficient GWG. However, it is unknown whether the IOM reference is adequate for Mexican pregnant adolescents, which is a public health concern as we currently do not have any official parameters to evaluate GWG in adolescent pregnancy. The number of LGA neonates was small (n = 20). Therefore, certain estimates were imprecise.

Although our sample was for convenience considering the inclusion criteria, we must consider that INPer is a national reference center that provides prenatal control for women from several regions of Mexico. Moreover, our study had a prospective follow-up.

## 5. Conclusions

To the best of our knowledge, this is the first study to analyze the association between maternal dietary and nutrient intake and eating habits and GWG and birth weight in a sample of pregnant adolescent–baby dyads. Furthermore, we show that when certain elements of the diet are inadequate, optimal maternal and neonatal outcomes can be limited. In addition, all models were adjusted by pBMI in order to control its confounding effect to a certain extent.

Pregnant adolescents need to know the relationship between the components of the diet and GWG to improve their eating habits. Health personnel should promote the consumption of a healthy diet according to the individual requirements of pregnant adolescents and promote avoidance of inappropriate eating habits while considering sociocultural and economic characteristics.

The consumption of adequate amounts of legumes, cereals and grains, animal-sourced foods, and sugar-sweetened beverages is part of the dietary guidelines because their consumption is related to health outcomes such as weight gain and diabetes. However, our study provides evidence of other health outcomes, such as GWG and birth weight in the studied group of pregnant adolescents, which could be affected by eating habits. Our results can inform the development of clinical and nutritional guidelines for antenatal control aimed at preventing complications and promoting healthy pregnancy.

## Figures and Tables

**Table 1:** Adolescents’ gestational weight gain and offspring’s birth weight according to dietary intake.

Food Group	Intake	Gestational Weight Gain (%)			Birth Weight (%)		
		Insufficient, n = 205	Excessive, n = 187	*p*-Value	SGA, n = 108	LGA, n = 20	*p*-Value
Vegetables	Adequate, n = 37	32.4	37.8	0.785	27.0	5.4	0.472
	Insufficient, n = 493	37.7	36.5		19.9	3.7	
Fruits	Adequate, n = 149	33.6	36.2	0.335	21.5	3.4	0.891
	Insufficient, n = 381	38.8	36.7		19.9	3.9	
Legumes	Adequate, n = 54	42.6	20.4	0.023	16.7	1.9	0.536
	Insufficient, n = 476	36.8	38.4		20.8	4.0	
Cereal and grains	Adequate, 7–11 n = 203	33.5	36.9	≤0.001	23.6	3.0	0.256
	Insufficient, <7 n = 211	53.1	20.4		20.4	3.3	
	Excessive ≥12 n = 116	15.5	65.5		14.7	6.0	
Animal-	Adequate, n = 118	31.4	39.0	≤0.001	22.0	3.4	0.941
source	Insufficient, n = 368	41.3	32.1		20.1	4.1	
foods	Excessive, n = 44	20.5	68.2		18.2	2.3	
Fats and	Adequate, n = 151	33.1	43.0	0.275	17.9	3.3	0.795
oils	Insufficient, n = 361	39.1	33.5		21.1	3.9	
	Excessive, n = 18	38.9	44.4		27.8	5.6	
Milk and	Adequate, n = 171	38.6	40.9	0.270	20.5	3.5	0.822
yogurt	Insufficient, n = 257	38.1	33.5		20.2	4.7	
	Excessive, n = 102	33.5	37.3		20.6	2.0	
Sugar table	Adequate, n = 226	39.8	33.6	0.442	18.6	4.0	0.671
	Excessive, n = 304	35.5	38.8		21.7	3.6	
Sugar-sweetened	Adequate, n = 171	33.3	33.3	0.030	14.6	4.7	0.066
beverage	Excessive, n = 359	39.3	38.2		23.1	3.3	
Number of	≥4, n = 106	29.2	40.6	0.151	18.9	2.8	0.754
food groups	≤3, n = 424	39.4	35.6		20.8	4.0	

Percentages estimated by rows. SGA: small for gestational age. LGA: large for gestational age. *p*-value determined by Pearson’s Chi-Square.

**Table 2:** Adolescent’s gestational weight gain and offspring’s birth weight according to energy and macronutrients intake.

Nutrient Intake	Gestational Weight Gain (%)			Birth Weight (%)		*p*-Value
	Insufficient, n = 205	Excessive, n = 187	*p*-Value	SGA, n = 108	LGA, n = 20	
Adequacy energy						
Adequate (80–120%) n = 248	35.9	38.3	0.577	18.5	3.6	0.736
Low (<80%), n = 147	37.4	32.7		19.7	4.1	
Excessive (>120), n = 135	40.7	37.8		24.4	3.7	
Carbohydrates						
Adequate (45–55%),n = 226	36.7	37.2	0.727	19.0	4.9	0.698
Low (<45%), n = 91	31.9	39.6		18.7	3.3	
Excessive (>55), n = 213	40.4	34.7		22.5	2.8	
Lipids						
Adequate (25–30%), n = 253	38.3	36.4	0.590	24.1	4.0	0.245
Low (<25%), n = 125	41.6	33.6		18.4	2.4	
Excessive (>30%), n = 152	32.2	39.5		15.8	4.6	
Proteins						
Adequate (15–20%), n = 227	38.3	35.2	0.881	24.2	3.5	0.379
Low (<15%), n = 225	35.6	39.1		17.3	4.4	
Excessive (>21%), n = 78	39.7	33.3		17.9	2.6	

Percentages estimated by rows. SGA: small for gestational age. LGA: large for gestational age. *p*-value determined by Pearson’s Chi-Square.

**Table 3:** Adolescents’ gestational weight gain and offspring’s birth weight according to eating habits.

Eating Habits	Gestational Weight Gain (%)			Birth Weight (%)		
	Insufficient, n = 205	Excessive, n = 187	*p*-Value	SGA, n = 108	LGA, n = 20	*p*-Value
Number of meals						
≥3, n = 121	35.5	34.7	0.695	23.1	3.3	0.855
3, n = 300	38.7	35.7		20.3	3.7	
≤2, n = 109	35.8	41.3		17.4	4.6	
Having breakfast						
Yes, n = 505	36.6	36.8	0.263	19.6	4.0	0.099
No, n = 25	52	32.0		36.0	0.0	
Having lunch						
Yes, n = 524	37.8	36.6	0.047	20.6	3.8	0.381
No, n = 6	0.0	33.3		0.0	0.0	
Having dinner-super						
Yes, n = 467	36.8	37.3	0.591	21.2	3.9	0.408
No, n = 63	42.9	31.7		14.3	3.2	
Skipping meals						
Never, n = 245	36.3	37.6	0.157	20.8	3.3	0.819
1–3 times/week, n = 222	40.1	32.0		21.2	4.5	
4–5 times/week, n = 63	31.7	49.2		15.9	3.2	
Eating out of home						
Yes, n = 73	32.9	34.2	0.349	20.6	3.7	0.954
No, n = 457	38.1	37.0		19.2	4.1	
Eating alone						
Yes, n = 107	35.5	43.0	0.262	19.6	5.6	0.535
No, n = 423	37.0	35.0		20.6	3.3	
Activities during the meals						
None, n = 205	36.6	34.6	0.793	22.4	1.5	0.190
Watching TV or using a cellphone, n = 270	37.4	38.5		18.5	5.6	
Doing household chores, n = 55	40.0	34.5		21.8	3.6	
Modify their feeding						
Was better, n = 353	36.3	36.5	0.544	19.8	3.4	0.644
Was worse, n = 103	38.8	40.8		23.3	5.8	
No change, n = 74	40.5	31.1		18.9	5.8	

Percentages estimated by rows. SGA: small for gestational age. LGA: large for gestational age. None of the variables was statistically significant. *p*-value determined by Pearson’s Chi-Square.

**Table 4:** Poisson regression models of adolescents’ gestational weight gain and offspring’s birth weight as outcome and dietary intake as predictors.

	Gestational Weight Gain				Birth Weight			
	Insufficient		Excessive		SGA		LGA	
	PR	95% CI	PR	95% CI	PR	95% CI	PR	95% CI
Legumes								
M1	1.16	0.75–1.79	**1.89**	**1.03–3.47**	–	–	–	–
M2	0.80	0.51–1.28	**1.95**	**1.05–3.60**	–	–	–	–
M3	0.82	0.52–1.28	**1.86**	**1.00–3.44**	–	–	–	–
Cereal and grains								
<7 servings								
M1	1.59	**1.17–2.14**	0.55	0.38–0.80	–	–	–	–
M2	1.61	**1.19–2.18**	0.55	0.38–0.80	–	–	–	–
M3	1.56	**1.14–2.12**	0.57	0.39–0.83	–	–	–	–
>12 Excessive								
M1	0.46	0.28–0.78	**1.77**	**1.29–2.44**	–	–	–	–
M2	0.47	0.28–0.79	**1.77**	**1.29–2.44**	–	–	–	–
M3	0.49	0.29–0.82	**1.65**	**1.18–2.29**	–	–	–	–
Animal–source foods								
Insufficient								
M1	1.32	0.92–1.89	0.82	0.59–1.16	–	–	–	–
M2	1.35	0.94–1.94	0.81	0.57–1.14	–	–	–	–
M3	1.43	0.99–2.05	0.72	0.51–1.02	–	–	–	–
Excessive								
M1	0.65	0.32–1.35	**1.75**	**1.04–2.77**	–	–	–	–
M2	0.70	0.34–1.46	**1.65**	**1.03–2.65**	–	–	–	–
M3	0.80	0.38–1.68	1.33	0.82–2.17	–	–	–	–
Consume sweetened beverages								
M1	1.18	0.87–1.60	1.15	0.84–1.56	1.58	**1.01–2.47**	0.71	0.29–1.75
M2	1.16	0.85–1.59	1.14	0.84–1.56	1.58	**1.00–2.47**	0.77	0.31–1.94
M3	1.19	0.87–1.62	1.14	0.84–1.56	1.58	**1.01–2.49**	0.78	0.31–1.98
≤3 Food groups								
M1	1.40	0.95–2.07	0.88	0.63–1.23	–	–	–	–
M2	1.31	0.89–1.93	0.90	0.64–1.27	–	–	–	–
M3	1.34	0.91–1.98	0.86	0.61–1.21	–	–	–	–

*p*-value determined by Poisson regression. PR: prevalence ratio; CI: confidence interval; SGA: small for gestational age. LGA: large for gestational age. M stands for Model: M1, crude; M2: adjusted by socioeconomic level, school drop-out, education, gynecological age, chronological age, and antenatal care; M3, adjusted by the same variables included in M2 plus pBMI. In bold are present the significant results.

**Table 5:** Poisson regression models of adolescents’ gestational weight gain and offspring’s birth weight as outcome and lipids intake and eating habits as predictors.

Lipids	Gestational Gain, %				Birth Weight			
	Insufficient		Excessive		Small		Large	
	PR	95% CI	PR	95% CI	PR	95% CI	PR	95% CI
Adequate REF								
Insufficient								
M1	–	–	–	–	0.70	0.46–1.08	0.55	0.14–2.21
M2	–	–	–	–	0.74	0.43–1.26	0.35	0.08–1.48
M3	–	–	–	–	0.74	0.44–1.27	0.35	0.08–1.52
Excessive								
M1	–	–	–	–	0.71	0.42–1.20	0.95	0.35–2.56
M2	–	–	–	–	0.73	0.47–1.26	0.86	0.31–1.49
M3	–	–	–	–	0.75	0.49–1.27	0.85	0.31–2.34
Skipping meals								
None REF								
1–3 time/week								
M1	1.10 *	0.82–1.48	0.85	0.63–1.16	–	–	–	–
M2	1.14	0.85–1.53	0.85	0.62–1.16	–	–	–	–
M3	1.15	0.85–1.54	0.82	0.60–1.12	–	–	–	–
4–5 time/week								
M1	0.87	0.54–1.42	1.31	0.87–1.97	–	–	–	–
M2	0.87	0.54–1.42	1.30	0.87–1.97	–	–	–	–
M3	0.92	0.57–1.51	1.17	0.77–1.77	–	–	–	–
Number of meals								
>3 REF								
3								
M1	1.01	0.69–1.47	1.09	0.73–1.62	–	–	–	–
M2	1.03	0.70–1.52	1.12	0.75–1.69	–	–	–	–
M3	1.03	0.70–1.52	1.08	0.72–1.64	–	–	–	–
<2								
M1	1.01	0.66–1.54	1.22	0.79–1.88	–	–	–	–
M2	1.04	0.68–1.59	1.23	0.80–1.90	–	–	–	–
M3	1.08	0.70–1.65	1.09	0.70–1.70	–	–	–	–
Activities during the meals								
Seeing TV								
M1	–	–	–	–	0.83	0.55–1.23	**3.80**	**1.10–13.11**
M2	–	–	–	–	0.84	0.56–1.26	**3.92**	**1.11–13.84**
M3	–	–	–	–	0.87	0.58–1.30	**3.76**	**1.06–13.36**
Doing household chores								
M1	–	–	–	–	0.97	0.52–1.84	2.49	0.42–14.87
M2	–	–	–	–	0.98	0.52–1.87	2.67	0.44–16.45
M3	–	–	–	–	0.98	0.52–1.86	2.89	0.46–18.07

* *p* <0.050. *p*-value determined by Poisson regression. PR: prevalence ratio; CI: confidence interval; SGA: small for gestational age. LGA: large for gestational age. M stands for model: M1, crude; M2, adjusted by socioeconomic level, school drop-out, education, gynecological age, chronological age, and antenatal care; M3, adjusted by the same variables included in M2 plus pBMI. In bold are present the significant results.

## Data Availability

The data presented in this study are available from the corresponding author upon reasonable request.

## Appendix A

Recommended intake and number of servings of food groups for adolescent mothers.

**Food Group**	**Recommendation**	**Real Intake ***
Vegetables	>3	0 (0–1)
Fruits	3–4	1.5 (0.5–3)
Grain and cereals	8–11	8 (6–11)
Legumes	2–2.5	0 (0–0)
Animal-source foods	3.5–4	3 (2–4)
Fat and oils	3–5	2 (1–3)
Milk and yogurt	2–2.5	2 (1–2)
Sugar table	<5	1 (0–2)
Sugar sweetened beverages	0	2 (0.5–11)
Academia Nacional de Medicina 2015. México (Fernández-Gaxiola et al. 2015). * Median (p25–75).		

## Appendix B

GWG of adolescent mother and offspring birth weights according to sociodemographic characteristics (%).

	**Gestational Weight Gain %**		***p*-Value**	**Birth Weight**	**LGA, n = 20**	***p*-Value**
	**Insufficient**	**Excessive**		**SGA,** **n = 108**		
Chronological age (years)						
≤15, n = 204	31.9	39.7	0.038	22.5	3.4	0.601
≥16, n = 326	42.9	32.5		19.0	4.0	
Beginning antenatal care						
First, n = 89	37.1	33.7	0.762	15.7	3.4	0.134
Second, n = 338	37.9	37.0		21.0	5.0	
Third, n = 103	42.7	31.1		22.3	0.0	
Marital status						
Single, n = 323	37.8	37.2	0.525	20.4	4.3	0.694
Cohabiting, n = 207	40.1	32.4		20.3	2.9	
Occupation						
Student, n = 57	42.1	36.8	0.655	19.3	1.8	0.668
Housewife, n = 473	38.3	35.1		20.5	4.0	
Socioeconomic level						
Middle, n = 143	38.5	37.1	0.464	18.9	2.8	0.292
Low, n = 213	34.7	37.1		17.8	3.3	
Very low, n = 174	43.7	31.6		24.7	5.1	
School dropout						
No, n = 193	37.3	38.3	0.526	19.2	3.6	0.858
Yes, n = 337	39.5	33.5		21.1	3.9	
Gestational age						
Term >37, n = 473	40.0	34.7	0.215	19.9	3.6	0.550
Preterm, n = 57	28.1	40.4		26.4	5.3	
Number of prenatal visits						
≤6, n = 437	41.6	33.9	0.459	19.7	3.4	0.877
≥7, n = 93	36.4	36.4		20.9	4.0	
Percentages estimated by rows. SGA: small for gestational age. LGA: large for gestational age. *p*-value determined by Pearson’s Chi-Square.						
